
JOURNAL INFORMATION
==============================
NLM Title Abbreviation: ScientificWorldJournal
Journal ID: ScientificWorldJournal
NLM Journal ID: 101131163
Journal ID: TSWJ

The Scientific World Journal

ISSN: 2356-6140
EISSN: 1537-744X
Publisher: Wiley

ARTICLE INFORMATION
==============================
PMCID: PMC5956458
PMID: 15349526
DOI: 10.1100/tsw.2004.45
Article ID: 702518
Article version: 1
Subjects: Original Report

Bladder Outflow Obstruction Caused by Prostatic Calculi

Calleja R.
Yassari R.
Wilkinson E.P.
Webb R.
Department of Urology, Norfolk and Norwich Hospital, Norfolk, UK
Academic Editor: Anthony Atala

Publication date: 2004
Electronic publication date: 2004 Jun 7
Volume: 4
First page: 46
Last page: 47
Copyright: Copyright © 2004 R. Calleja et al.
Copyright year: 2004
License: This is an open access article distributed under the Creative Commons Attribution License, which permits unrestricted use, distribution, and reproduction in any medium, provided the original work is properly cited.
License URL: https://creativecommons.org/licenses/by/3.0/

==============================
Original Report
*TheScientificWorldJOURNAL* (2004) 4 (S1), 46–47
ISSN 1537-744X; DOI 10.1100/tsw.2004.45

### Bladder Outflow Obstruction Caused by Prostatic Calculi

R. Calleja, MSc (Urol), FRCS (Urol), R. Yassari, MD, E.P. Wilkinson, BSc, MBBS, and R. Webb, MS, FRCS (Urol)
*Department of Urology, Norfolk and Norwich Hospital, Norfolk, UK.*

*Previously published in the Digital Urology Journal*

**DOMAIN**: urology

### CASE REPORT

A 76 year-old male presented with worsening symptoms of bladder outflow obstruction, with a progressive history of hesitancy, worsening stream and terminal dribbling. Routine pre-operative investigations did not reveal any significant abnormality except fora plain abdominal X-Ray, that showed considerable prostatic calcification (Figure). At cystoscopy, he was found to have an occlusive looking prostate and the prostatic urethra had a calculus embedded in it.

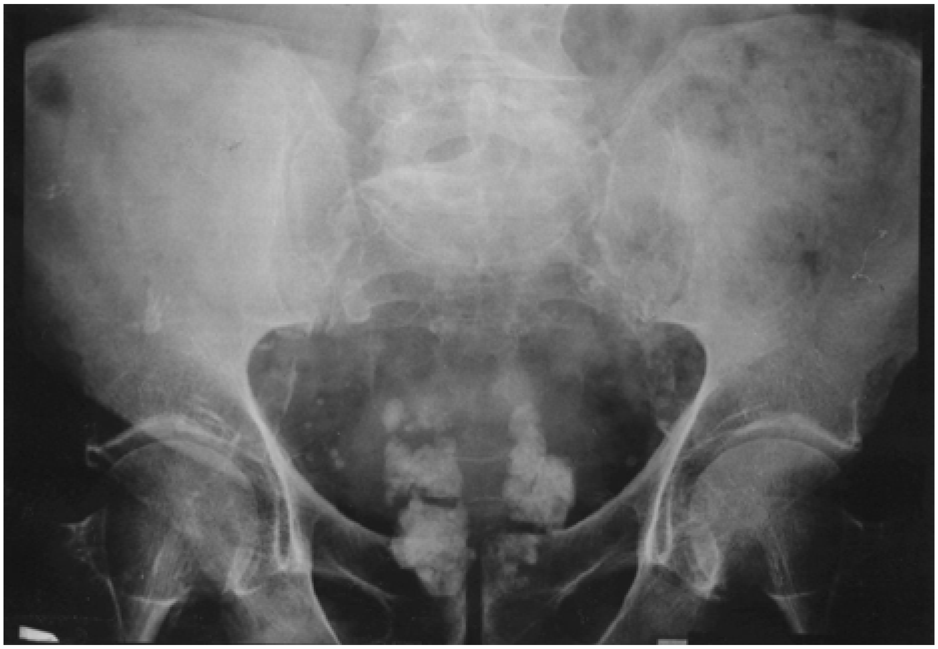

A transurethral resection of the prostate (TURP) was performed and only 8 grams of tissue were resected. Five large calculi filling up lacunae just below the prostatic surface were found and removed. Histology subsequently showed Chronic Focal Prostatitis.

The patient re-presented after 6 weeks with retention of urine. Further cystoscopy revealed large amounts of calculi in the bladder, urethra and in the prostatic cavity. An additional 50 grams of prostatic calculi were subsequently extracted and the patient was once again able to void normally.

### DISCUSSION

Prostatic calculi are extremely common in men over 50years of age but infrequent in patients below 40 years and rare in children.

Prostatic calculi are usually composed of calcium phosphatestones[1] formed either by simple precipitation of prostatic secretions or calcification of the corpora amylacea. They may arise either spontaneously or as a result of inflammation, infection or obstruction.[2]

99% of all prostates examined at autopsy[2,3] show calculi mainly in the border zone between the middle and postero-lateral lobes of the prostate. Prostatic calculi appear to be unrelated to the development of adenocarcinoma, but if calculi are present, they are typically seen peripheral to the tumors.

Symptoms attributed to prostatic calculi are rare and include reduction of the urinary stream, lower back and leg pain,[2] recurrent passage of calculi after TURP and orchitis.[4]

Prostatic calculi rarely present as a clinical problem and their diagnosis is usually obtained by plain radiology or transrectal ultrasonography.[5] Treatment of the calculi is often not required, but usually involves transurethral resection although ESWL with suprapubic percutaneous extraction[6] has been described.

Large prostatic calculi detected pre-operatively in the presence of small amounts of resectable prostatic tissue should be treated and extracted as aggressively and completely as possible at the first intervention to avoid the necessity of repeat intervention.

### REFERENCES

1. Gawande, A.S.: Brushite Lithiasis of Prostate. B. J. Urol 58 (2): 230, 1986.
2. Klimas R., Bennett. B., Gardner WA Jr. Prostatic Calculi: A review. Prostate; 7(1) :91, 1985.
3. Sondergaard G, Vetner M. , Christensen PO: Prostatic Calculi. Acta Pathol. Microbiol. Immunol. Scand; 95(3) : 141-5,1987.
4. Koh K.B.: Symptomatic Prostatic Calculi- A Rare Complication After TURP. Med. J Malaysia. ; 50 (3) : 280-1, 1995.
5. Dahnert WF. , Hamper UM., Walsh PC., Eggleston JC., Sanders RC.: The Echogenic Focus In Prostatic Sonograms, With Xeroradiographic And Histopathologic Correlation. Radiology.; 159(1): 95-100. 1986
6. Melone F., Lardani T., Azzaroli G. et al.: Dumbell Stone Of Prostatic Fossa After Prostatectomy. A Combined ESWL And Suprapubic Percutaneous Treatment. Acta Urologica Belgica., 64(4) : 27-31, 1996.

**This article should be referenced as follows:**

Calleja, R., Yassari, R., Wilkinson, E.P., and Webb, R. (2004) Bladder outflow obstruction caused by prostatic calculi. *TheScientificWorldJOURNAL* **4 (S1)**, 46–47.

**Handling Editor:**

Anthony Atala, Principle Editor for *Urology* — a domain of *TheScientificWorldJOURNAL*.
